# Insight on the Structure-to-Activity of Carbosilane Metallodendrimers in the Fight against *Staphylococcus aureus* Biofilms

**DOI:** 10.3390/antibiotics10050589

**Published:** 2021-05-17

**Authors:** Celia Llamazares, Natalia Sanz del Olmo, Juan Soliveri, F. Javier de la Mata, José Luis Copa-Patiño, Sandra García-Gallego

**Affiliations:** 1 University of Alcala, Department of Biomedicine and Biotechnology, 28805 Madrid, Spain; celia.llamazares@edu.uah.es (C.L.); juan.soliveri@uah.es (J.S.); josel.copa@uah.es (J.L.C.-P.); 2 University of Alcala, Research Institute in Chemistry “Andrés M. del Río” (IQAR) and Faculty of Science, Department of Organic and Inorganic Chemistry, 28805 Madrid, Spain; natalia.sanzo@edu.uah.es (N.S.d.O.); javier.delamata@uah.es (F.J.d.l.M.); 3 Networking Research Center on Bioengineering, Biomaterials and Nanomedicine (CIBER-BBN), 28029 Madrid, Spain; 4 University of Alcala, Institute Ramón y Cajal for Health Research (IRYCIS), 28034 Madrid, Spain

**Keywords:** dendrimer, metallodendrimer, metal, copper, ruthenium, antibacterial, biofilm, *Staphylococcus aureus*

## Abstract

Biofilm formation is a critical health concern, involved in most human bacterial infections. Combatting this mechanism, which increases resistance to traditional antibiotics and host immune defences, requires novel therapeutic approaches. The remarkable biocide activity and the monodispersity of carbosilane metallodendrimers make them excellent platforms to evaluate the impact of different structural parameters on the biological activity. In this work, we explore the influence of iminopyridine ring substituents on the antibacterial activity against planktonic and biofilm *Staphylococcus aureus*. New families of first-generation Ru(II) and Cu(II) metallodendrimers were synthesised and analysed, in comparison to the non-substituted counterparts. The results showed that the presence of methyl or methoxy groups in *meta* position to the imine bond decreased the overall positive charge on the metal ion and, subsequently, the activity against planktonic bacteria. However, it seemed a relevant parameter to consider for the prevention of biofilm formation, if they contribute to increasing the overall lipophilicity. An optimum balance of the charge and lipophilicity of the metallodrug, accomplished through structural design, will provide effective biocide agents against bacteria biofilms.

## 1. Introduction

*Staphylococcus aureus* are Gram-positive bacteria which represent a serious health issue. *S. aureus* belongs to the *Staphylococcaceae* family and appears in coconut shape with 0.5 to 1.5 microns diameter, arranged in pairs, clusters or chains [1]. The widespread dissemination of this microorganism, together with its virulence and antibiotic resistance, produces an important impact on morbidity at community and intra-hospital levels [2]. This partly arises from its ability to adhere to the surface of permanent medical devices and develop biofilms [3]. Biofilms represent an evolved system that enables bacteria to survive in hostile environments, forming permanent colonies prone to dissociate and form new colonies [4,5,6]. Biofilms are up to 1000 times more resilient to the treatment with conventional antibiotics, compared to planktonic bacterial growth [7]. All these circumstances increase the survival capacity of *S. aureus* and the situation is aggravated by the multiple antibiotic resistance developed by the pathogen, hindering the treatment of diseases caused by these bacteria [8].

Nanotechnology provides innovative solutions in the fight against infectious diseases, including biofilm-forming bacteria [9]. Metal nanoparticles, carbon-based nanomaterials, liposomes, polymeric nanoparticles and dendrimers with antimicrobial activity have been reported in the literature. In particular, dendrimers and dendritic materials are sophisticated tools in the treatment, prevention and diagnosis of highly prevalent infectious diseases [10,11]. They offer advantages such as the highly branched globular conformation, the monodispersity and the multivalent decoration on their surface. Indeed, most antimicrobial dendrimers—peptidic, glycodendrimers, quaternary ammonium decorated—exploit the multivalence to efficiently bind to the negatively charged bacteria membrane or the membrane components and subsequently causing cell membrane disruption.

The antimicrobial action can be further improved by the presence of metal ions in the nanoparticle, which can damage microbial cells through membrane degradation, protein dysfunction and oxidative stress [12]. So far, a very few examples of antimicrobial metallodendrimers have been reported in the literature [13]. Our group recently confirmed the outstanding antibacterial activity of iminopyridine carbosilane dendrimers bearing Ru(II) and Cu(II) complexes [14]. The presence of the hydrophobic, stable and flexible carbosilane scaffold enhances the interaction with biological membranes and improves the overall biological activity [15]. Parameters such as the dendrimer generation, the nature of the metal ion or the ligands on the metal enabled a fine-tuning of the bacteriostatic and bactericide activity. An additional parameter—the substituents on the iminopyridine ring- has recently proved to be key for the biological activity of these metallodendrimers [16]. Electron-donating substituents, such as methyl and methoxy, in *para*- position to the pyridine nitrogen and in *meta*- position to the imine group, significantly increased the antitumor properties towards myeloid cancer cells. Surprisingly, this behaviour was only observed if the inductive and resonant properties of the substituent were paired with the right metal counterion. The methyl/chloride and methoxy/nitrate pairs unveiled potent antitumor activities of Cu(II) metallodendrimers, but through two different mechanisms of action. This exemplifies the importance of adequately designing the dendritic scaffold to maximise the biological activity.

Encouraged by this revelation, we herein analyse the influence of this parameter—the substituents on the iminopyridine ring—with the final aim of improving the antimicrobial activity against biofilm-forming bacteria. We present a new family of Ru(II) and Cu(II) metallodendrimers bearing R substituents in *meta*- position to the imine bond, as an alternative to traditional antibiotics. Bacteriostatic and bactericide activity against *S. aureus* is tested, in both planktonic and biofilm forms, and compared to the non-substituted counterparts. A useful structure-to-activity insight is provided for the designing of effective antibacterial agents.

## 2. Results

**Figure 1:**
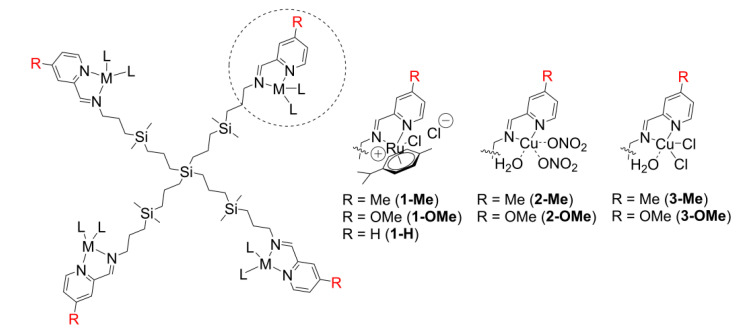
Chemical representation of the Ru(II) and Cu(II) carbosilane metallodendrimers, bearing R-substituents in *meta* position to the imine moiety.

A library of compounds was selected to evaluate the impact of the ring substituent as well as the nature of the metal and the ligands. Considering the promising results obtained in our former studies, only first generation metallodendrimers were targeted in this work. The selected families comprised ruthenium (II) metallodendrimers with cymene ligand G_1_-[[NCPh(*o*-N)(*m*-R)Ru(η^6^-*p*-cymene)Cl]Cl]_4_ (**1-R**), and copper (II) metallodendrimers with nitrate G_1_-[NCPh(*o*-N)(*m*-R)Cu(ONO_2_)_2_·H_2_O]_4_ (**2-R**) and chloride G_1_-[NCPh(*o*-N)(*m*-R)CuCl_2_·H_2_O]_4_ (**3-R**) ligands (Figure 1). The iminopyridine substituent R was H, Me and OMe. The Ru(II) metallodendrimer with R=H and all the Cu(II) metallodendrimers were synthesised according to the published protocols [16,17].

### 2.1. Synthesis and Characterisation of Ru(II) Metallodendrimers with R-Substituted Iminopyridine Rings

**Scheme 1:**
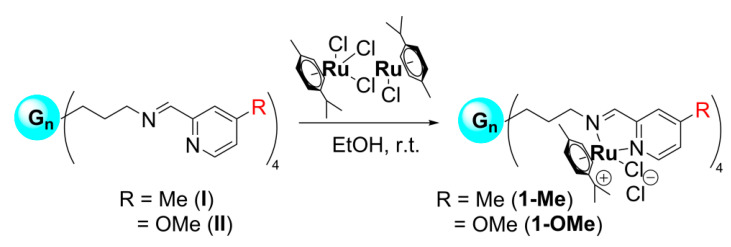
Synthesis of new Ru(II) metallodendrimers bearing R-substituted iminopyridine rings.

The synthesis of the new Ru(II) metallodendrimers followed a parallel route to the one leading to G_1_-[[NCPh(*o*-N)(*m*-H)Ru(η^6^-*p*-cymene)Cl]Cl]_4_ (**1-H**) [17]. In this case, the dendrimers G_1_-[NCPh(*o*-N)(*m*-CH_3_)]_4_ (**I**) and G_1_-[NCPh(*o*-N)(*m*-OCH_3_)]_4_ (**II**) [16] were used as precursors, Scheme 1. The addition of [Ru(η^6^-*p*-cymene)Cl_2_]_2_ to the dendrimer solution in ethanol in a ratio 1:1 branch:[Ru] led to the formation of the metallodendrimers G_1_-[[NCPh(*o*-N)(*m*-Me)Ru(η^6^-*p*-cymene)Cl]Cl]_4_ (**1-Me**) and G_1_-[[NCPh(*o*-N)(*m*-OMe)Ru(η^6^-*p*-cymene)Cl]Cl]_4_ (**1-OMe**), respectively. Both metallodendrimers were characterised by ^1^H-NMR, ^13^C-NMR and elemental analysis. The analytical and spectroscopic data (see Experimental Section and Supplementary Material) confirmed the presence of the structures shown in Figure 1.

The metal coordination to the iminopyridine groups in the precursors **I** and **II** was confirmed by the shifting of the signals to higher frequency in ^1^H-NMR and ^13^C-NMR spectra. Table 1 summarises the most significant shifts in ^1^H-NMR and further details can be found in the Supporting Information. The pyridine signals, which appear at 7.1–8.5 ppm for the methyl-precursor **I** and at 6.8–8.4 ppm for the methoxy-precursor **II**, are significantly shifted, up to +0.8 ppm for the proton in *meta*- position. The imine group –CH=N shifts +0.30 ppm and the adjacent methylene groups undergo a significant change: besides the important shift, two sets of signals appear for each methylene group.

### 2.2. Surface Charge of Ru(II) Metallodendrimers

In order to characterise the surface charge of the Ru(II) metallodendrimers, conductivity and zeta potential measurements were performed. For an accurate comparison, samples were tested at the same concentration. The results from both techniques concluded that the selected metallodendrimers exhibited cationic properties (Table 2). The highest conductivity (160.2 µS/cm) was obtained with **1-H**, followed by the substituted counterparts **1-OMe** and **1-Me**. The high Z-potential values in the range 50.2–61.2 mV also confirmed the overall cationic charge in the molecule. Compared to the analogous Ru(II) metallodendrimer with Cp/PTA ligands **4-H**, the compounds bearing cymene ligands duplicated the Z-potential values.

### 2.3. Antibacterial Activity of Ru(II) Carbosilane Metallodendrimers Against Planktonic and Biofilms S. aureus

In the previous work, Ru(II) carbosilane metallodendrimes had shown a potent antibacterial activity in planktonic cells, which even surpassed the activity of Cu(II) analogues. However, the bacteriostatic and bactericide effect decreased when *S. aureus* are prompt to form biofilm. Herein, the antibacterial behaviour of Ru(II) metallodendrimers **1-R** was evaluated towards *Staphylococcus aureus* as a model of Gram-positive bacteria. Two different experiments were performed: First, the activity towards planktonic *S. aureus.* Second, the activity to prevent the formation of *S. aureus* biofilm. Table 2 summarises the obtained values for the minimum inhibitory concentration (MIC) and the minimum bactericidal concentration (MBC) for each Ru(II) complex, as well as minimum biofilm formation inhibitory concentration (MBIC) and the minimum bactericidal concentration for biofilms formation (MBC-B).

The data shown in Table 2 indicate that Ru(II) metallodendrimers bearing cymene ligands are moderate antibacterial agents towards *S. aureus.* These metallodendrimers, with four metal atoms in their periphery, displayed planktonic MIC values in the range 16–64 mg/L for *S. aureus*. The highest activity is found for **1-H**, followed by **1-OMe** and **1-Me**. Regarding the nature of the ligands in the Ru(II) complex, the presence of cymene clearly reduced the efficacy of the metallodrug, compared to the Cp/PTA analogue which had an excellent MIC value of 4 mg/L. Nevertheless, the impact of the ring substituent and the ligand was observed in *S. aureus* biofilms. In this assay, similar values were obtained for the four Ru(II) metallodendrimers tested. The moderate values of **4-H** (MBIC = 32 mg/L and MBC-B = 128 mg/L) were even slightly improved, as observed in **1-Me** (MBIC = 16 mg/L and MBC-B = 64 mg/L).

### 2.4. Antibacterial Activity of Cu(II) Carbosilane Metallodendrimers Against Planktonic and Biofilms S. aureus

**Figure 2:**
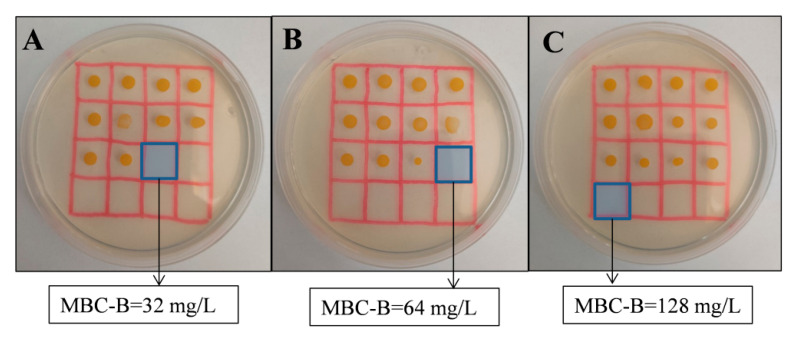
Effect of R-substituted metallodendrimers in preventing the formation of *S. aureus* biofilms. (**A**) Compound **3-OMe**, with Cu(II) chloride complex; (**B**) compounds **2-OMe** and **1-Me**, with Cu(II) nitrate and Ru(II) cymene complexes, respectively; and (**C**) compounds **2-Me**, **3-Me** and **1-OMe**, with Cu(II) nitrate, Cu(II) chloride and Ru(II) cymene complexes, respectively. The minimum bactericidal concentration for biofilms (MBC-B) represents the minimal concentration of the metallodendrimer that can kill the microorganism in the conditions used to produce a biofilm.

While Ru(II) metallodendrimers are moderate candidates, the Cu(II) counterparts have shown potent bacteriostatic and bactericide activity in both planktonic and biofilm *S. aureus*. In order to explore the impact of the substituents on the iminopyridine ring, we performed a similar study to that described for Ru(II) metallodendrimers. The results are summarised in Table 3 and Figure 2.

In planktonic cells, non-substituted metallodendrimers **2-H** and **3-H** had previously shown outstanding activity (MIC = 4 and 2 mg/L, respectively). As data on Table 3 indicate, the presence of ring substituents increased the MIC values in the trend H < OMe < Me. Furthermore, as previously observed, those complexes with chloride ligands produced a higher activity than the nitrate analogues. For biofilm-forming bacteria, however, we had observed that **2-H** kept the potent activity but the ligand exchange to chloride in **3-H** reduced the activity. This behaviour is replicated for the substituted counterparts: the nitrate-bearing complexes exhibited similar activity in planktonic and biofilm bacteria, while the chloride-ones are less efficient in biofilms. Again, the trend H < OMe < Me is generally observed for all the parameters tested.

## 3. Discussion

Metallodendrimers represent an attractive alternative to traditional antibiotics in the treatment of bacterial infections. Besides their promising bactericide and bacteriostatic activity, they offer valuable information about the impact of structural parameters on the therapeutic activity. The carbosilane metallodendrimers included in this study, together with those previously described [14], shape a useful library to analyse structure-to-antimicrobial activity relationships of the metallodrug.

A competitive antimicrobial candidate must be produced through a simple and cost-efficient process. Even though dendrimers are known for their tedious synthetic process [18], carbosilane scaffolds offer important advantages: the low generation dendrimers show outstanding biological activity, thus reducing the length and costs of their preparation. For the present study, only first generation metallodendrimers—bearing four metal complexes on the periphery—were selected, as they had shown the most promising activity in former assays [14]. The family of cymene-Ru(II) metallodendrimers **1-R** herein reported only requires a one-step synthetic route, under mild conditions and straightforward purification. This means a significant improvement from the 3-step route is required for cyclopentadienyl analogues. The R-substituted Cu(II) metallodendrimers had also proved technological advantages in the synthetic process, compared to the non-substituted analogues [16].

The coordination of the Ru(II) ions to the dendritic skeletons can be easily monitored through NMR spectroscopy (Table 1). We observed important shifts of the signals to higher frequency due to the effect of the electron-withdrawing metal. Furthermore, after the metal chelation, the cymene ring undergoes a loss of symmetry and generates a chiral molecule. This stereogenic centre induces a diastrophic effect around –CH_2_N and the methylene groups adjacent to the imine appear as two sets of signals. This effect has been previously described for the metallodendrimer **1-H** [17].

Most antibacterial dendrimers rely on an adequate cationic charge distribution and amphiphilicity to disrupt the negatively charged microbe membrane [10,19,20]. According to the conductivity and Z-potential results (Table 2), the metallodendrimers are positively charged and can disrupt bacterial membranes via electrostatic interactions or through exchange of the divalent metal ions which support the membrane. This non-specific mechanism reduces the possibilities to develop antibiotic resistance [11]. Among the Ru(II) complexes, the conductivity assays revealed that the cationic features decreased in the trend H > OMe > Me. The substituents in 4-position to the pyridine nitrogen induce a different basicity of this atom: while the 4-methyl group induces a +I effect, the 4-methoxyl generates a predominant +M effect which delocalises the negative charge in the nitrogen atom. Both increase the basicity of the nitrogen atom towards Lewis acids such as Ru(II) and Cu(II), thus reducing the overall positive charge on the metal ion compared to the non-substituted **1-H**, **2-H** and **3-H**. This confirms the crucial role of the metal complex in the antibacterial activity and the impact of modifying the type of metal or ligands in the complex. Furthermore, the Ru-cymene family **1-R** showed a good colloidal stability according to the Z-potential measurements, duplicating the values of the Ru-cyclopentadienyl derivative **4-H** and the Cu metallodendrimers **2-H** and **3-H**, with incipient instability [21]. The charge and colloidal stability of the metallodrug will also affect the antibacterial activity.

The antibacterial effect of the R-substituted metallodendrimers was tested using *S. aureus* as an example of Gram-positive bacteria, measuring the bacteriostatic and bactericide properties in planktonic cells (MIC, MBC) and biofilm-forming bacteria (MBIC, MIC, MBC-B). The assays confirmed the impact of the structural parameters—nature of the metal ion, ligands on the metal complex and substituents on the iminopyridine ring—on the antimicrobial activity

Ru(II) carbosilane metallodendrimers had previously shown a potent antibacterial activity in planktonic cells (MIC = 4 mg/L), which unfortunately decreased towards biofilm-forming bacteria [14]. The new family **1-R**, with cymene ligands in the ruthenium ion, does not improve the excellent activity in planktonic *S. aureus* and appear as moderate antibacterial agents (Table 2). Surprisingly, and despite the higher Z-potential of the cymene-bearing **1-H** compared to the Cp-bearing **4-H**, it shows lower activity (MIC = 16 mg/L). The hydrophobicity and stability provided by the Cp-ring together with the water-solubility and pH-responsiveness from the PTA ligand seem relevant for the antibacterial effect. A negative influence of the iminopyridine substituent is observed, decreasing the activity with the trend H > OMe > Me, in agreement with the loss of positive charge observed through conductivity assays. Nevertheless, the presence of the ring substituent might be meaningful towards biofilms: the metallodendrimer **1-Me** slightly improves the bacteriostatic and bactericide effect of ruthenium counterparts against biofilms. *S. aureus* biofilm formation occurs through diverse adherence mechanisms, based on polysaccharides, protein/eDNA, fibrin or amyloid proteins [22]. The increased lipophilicity (+π) in **1-Me** due to the methyl group may be responsible for the improvement in the preventive effect against biofilms, avoiding the adsorption of macromolecules to the surfaces and thus the formation of the extracellular polymeric matrix. Similarly, the increased hydrophobicity on cotton fabric after the deposition of Cu(II) poly(propylene imine) metallodendrimers prevented the formation of *B. cereus* and *P. aeruginosa* biofilms [23]. Both PPI and iminopyridine carbosilane dendrimers exhibit metal-chelating moieties in their scaffolds. The metal chelation reduces the polarity of the M(II) ion, increases the delocalisation of electrons over the chelate ring and increases the overall lipophilicity, favouring the penetration into the lipid cell membrane and overall improving the antibacterial effect. This highlights the importance of correctly balancing the charge and lipophilicity of the metallodrug.

Cu(II) carbosilane metallodendrimers had already shown potent activity in both planktonic *S. aureus* (MIC = 2–4 mg/L) and biofilms (MBIC = 4–8 mg/L) [14]. As data on Table 3 indicates, the presence of ring substituents increased the MIC values in the trend H < OMe < Me and again confirmed the negative effect on the antibacterial activity. This trend is generally observed for all the parameters tested. Regarding the metal ligands, chloride produced a higher activity than nitrate in planktonic cells, but slightly lost the activity in biofilms. This behaviour had also been observed for the non-substituted counterparts **2-H** and **3-H** [14]. Unfortunately, no substituent/counterion pairs were detected which clearly improved the antibacterial action. This crossed influence of the substituent/counterion substantially increased the antitumor activity of **3-Me** and **2-OMe** due to a remarkable production of reactive oxygen species (ROS) in U937 tumour cells, while barely affecting healthy PBMCs [16]. This confirms the different mechanism of action to produce the antitumor and antimicrobial effects. For the antimicrobial activity, it could be interesting to explore the effect of alternative ring substituents such as F, Cl or NO_2_, with +π and +σ properties [24], which may increase the overall lipophilicity and charge on the metallodrug.

We herein demonstrated that carbosilane metallodendrimers are a promising tool in the fight against Gram-positive bacteria, which cause important nosocomial infections in hospital facilities [2]. The presence of a thick peptidoglycan layer in these bacteria hampers the effective treatment and other dendrimers, such as PAMAM, reported difficulties to disrupt the crosslinked peptidoglycan layer [25]. Other metallodendrimers reported in the literature also showed antimicrobial effect towards planktonic *S. aureus* [13]. For example, a G2 aromatic polyamide dendrimer decorated with 6 Pd(II) ions exhibited a moderate MIC = 82 mg/L, but comparable to the antibiotic streptomycin (MIC= 80 mg/L) [26]. All carbosilane metallodendrimers described are far more effective, with MIC values ranging from 2 to 64 mg/L. This confirms the potential of our nanomaterials as an alternative to traditional antibiotics. In another example, a G1 propyletherimine dendrimer decorated with 6 Ag(I) carboxylate moieties showed MIC = 41.7 mg/L for *S. aureus* and 26.0 mg/L for methicillin-resistant *S. aureus* (MRSA) [27]. In this work, the authors demonstrated a direct relation between the number of Ag(I) ions and the antibacterial activity. Conversely, we have extensively demonstrated that first-generation carbosilane metallodendrimers are the most promising candidates and the G2 counterparts, which duplicate the number of metal ions, do not proportionally improve the activity [14,17]. The hydrophilic/hydrophobic balance in these metallodendrimers is crucial, and the overall antimicrobial activity is dictated not only by the metal, but also by the nature of the dendritic scaffold.

It is worth mentioning that none of the other metallodendrimers reported in the literature evaluated the effect towards biofilm-forming *S. aureus*, even though it is responsible for most human infections where *S. aureus* is involved. As we have proved, substantially different results may be obtained with the same metallodrug against planktonic and biofilm bacteria. For example, the Ru-cymene metallodendrimer **1-Me** exhibited the lowest positive charge and antibacterial activity in planktonic cells among the Ru(II) complexes; however, it presented a promising preventive activity against biofilms (MBIC= 16 mg/L and MBC-B = 64 mg/L). This reveals the need for a thorough antibacterial study, covering both planktonic and biofilm-forming bacteria, and the importance of fine-tuning the compound structure in order to maximise the efficiency to the resistant biofilms. The aim is finding a potent biocide activity towards both modes of growth, such as that exerted by the Cu-nitrate metallodendrimer **2-H** (MIC = MBIC = 4 mg/L) [14]. The higher cationic charge and lower membrane stabilisation of **2-H** [15] may explain the potent biocide activity. Further studies are required to evaluate the capacity of these metallodendrimers to disrupt the formation of already formed biofilms.

The complete study on carbosilane metallodendrimers revealed that these metallodrugs merged different mechanisms of action to attain the overall antibacterial effect. The cationic charge and the lipophilicity of the molecule [16], together with underlying mechanisms from the metal ions, such as the production of reactive oxygen species, are responsible for the overall antibacterial effect [28]. These must be adequately balanced for a correct activity in biofilms.

## 4. Materials and Methods

### 4.1. Metallodendrimers

Solvents were purified from appropriate drying agents. Chemicals were purchased from commercial sources and used as received. Elemental analyses were performed on a LECO CHNS-932. NMR spectra were recorded on a Varian Unity VXR-300 Hz and 500 Hz instruments. Chemical shifts are given in ppm. ^1^H and ^13^C resonances were measured relative to internal deuterated solvents peaks. {^1^H-^13^C}-HSQC-2D-NMR experiments were carried out to support the assignment of the signals.

#### 4.1.1. Synthesis and Characterisation of R-Substituted Iminopyridine Ru(II) Metallodendrimers

**G_1_-[[NCPh(*o*-N)(*m*-Me)Ru(η^6^-*p*-cymene)Cl]Cl]_4_ (1-Me).** [Ru(η^6^-*p*-cymene)Cl_2_]_2_ (35.0 mg, 0.0572 mmol) was slowly added to a solution of the first-generation dendrimer G_1_-[NCPh(*o*-N)(*m*-Me)]_4_ (30.7 mg, 0.0286 mmol) in dry ethanol. The solution was stirred overnight at room temperature. Subsequently, the solvent was evaporated and the metallodendrimer 1-Me isolated as brown solid (60.1 mg, 92%).

^1^H-NMR (CD_3_OD): δ (ppm) = 0.03 (s, 24H, -(C*H*_3_)_2_Si); 0.64 (m, 24H, -SiC*H_2_*); 1.03&1.17 (2d, 24H, (C*H_3_*)_2_CH^cym^); 1.39 (br m, 8H, -SiCH_2_C*H_2_*CH_2_Si-); 1.94 & 2.04 (2 m, 8H, -C*H_2_*CH_2_N); 2.28 (s, 12H, -CH_3_^cym^); 2.61 (s, 12H, CH_3_^Pyr^); 2.68 (m, 4H, -(CH_3_)_2_C*H*^cym^); 4.24&4.68 (2m, 8H, -C*H_2_*N); 5.83–5.79 (m, 8H, Ar^cym^); 6.05 (m, 4H, Ar^cym^); 6.17 (m, 4H, Ar^cym^); 7.64 (br s, 4H, Ar^pyr^); 8.00 (s, 4H, Ar^pyr^); 8.62 (s, 4H, -CH^imine^); 9.30 (s, 4H, Ar^pyr^); ^13^C {^1^H} NMR (CD_3_OD): δ (ppm) = −3.0 (-(*C*H_3_)_2_Si); 13.7 (-SiCH_2_*C*H_2_CH_2_Si); 18.6, 19.1, 19.8 (-Si*C*H_2_); 21.1 (-*C*H_3_^cym^ & -*C*H_3_^pyr^); 21.9, 23.0 (-(*C*H_3_)_2_CH^cym^); 25.8 (-SiCH_2_*C*H_2_CH_2_N); 32.4 (-(CH_3_)_2_*C*H^cym^); 71.5 (-*C*H_2_N); 85.5, 86.2, 86.8, 88.7 (-*C*H^cym^); 104.2, 106.6 (*C*^cym^); 130.3, 130.6 (*C*^pyr^); 154.0, 155.8, 156.2 (*C*H^pyr^); 168.4 (*C*H^imine^). Elemental Analysis (%): Calc. For C_100_H_156_Cl_8_N_8_Ru_4_Si_5_ (2298.70): C, 52.25; H, 6.84; N, 4.87; Found: C, 52.63; H, 7.24; N, 4.79.

**G_1_-[[NCPh(*o*-N)(*m*-OMe)Ru(η^6^-*p*-cymene)Cl]Cl]_4_ (1-OMe).** [Ru(η^6^-*p*-cymene)Cl_2_]_2_ (36.5 mg, 0.0596 mmol) was slowly added to a solution of the first-generation dendrimer G_1_-[NCPh(*o*-N)(*m*-OMe)]_4_ (33.9 mg, 0.0298 mmol) in dry ethanol. The solution was stirred overnight at room temperature. Subsequently, the solvent was evaporated and the metallodendrimer 1-OMe isolated as brown solid (67.2 mg, 96%).

^1^H-NMR (CD_3_OD): δ (ppm) = 0.04 (s, 24H, -(C*H*_3_)_2_Si); 0.62 (m, 24H, -SiC*H_2_*); 1.05&1.19 (2d, 24H, (C*H_3_*)_2_CH^cym^); 1.39 (br m, 8H, -SiCH_2_C*H_2_*CH_2_Si-); 1.93 & 2.04 (2 m, 8H, -C*H_2_*CH_2_N); 2.27 (s, 12H, -CH_3_^cym^); 2.69 (m, 4H, -(CH_3_)_2_C*H*^cym^); 4.05 (s, 12H, OCH_3_^Pyr^); 4.24&4.67 (2m, 8H, -C*H_2_*N); 5.77–5.81 (2s, 8H, Ar^cym^); 6.02 (s, 4H, Ar^cym^); 6.15 (s, 4H, Ar^cym^ ); 7.34 (br s, 4H, Ar^pyr^); 7.75 (s, 4H, Ar^pyr^); 8.59 (s, 4H, -CH^imine^); 9.22 (s, 4H, Ar**^pyr^**); ^13^C{^1^H} NMR (CD_3_OD): δ (ppm) = −2.9 (-(*C*H_3_)_2_Si); 13.7 (-SiCH_2_*C*H_2_CH_2_Si); 18.6, 19.1, 19.8 (-Si*C*H_2_); 21.1 (-*C*H_3_^cym^); 21.9, 23.0 (-(*C*H_3_)_2_CH^cym^); 25.8 (-SiCH_2_*C*H_2_CH_2_N); 32.4 (-(CH_3_)_2_*C*H^cym^); 57.6 (OCH_3_); 71.4 (-*C*H_2_N); 85.2, 85.9, 86.4, 88.4 (-*C*H^cym^); 103.8, 106.3 (*C*^cym^); 115.2, 116.6 (*C*^pyr^); 157.4, 157.5, 168.5 (*C*H^pyr^); 169.7 (*C*H^imine^). Elemental Anal. (%): Calc. For C_100_H_156_Cl_8_N_8_O_4_Ru_4_Si_5_ (2362.70): C, 50.84; H, 6.66; N, 4.74; Found: C, 51.17; H, 6.86; N, 4.70.

#### 4.1.2. Preparation of Metallodendrimers Samples

Serial dilutions of the different biocides were prepared in sterile distilled water for ruthenium complexes **1-R** and copper complexes with nitrate ligands **2-R** and due to their good solubility, and in DMSO:water (1:99 at the highest concentration) for chloride copper complexes **3-R**. The tested concentrations ranged from 0.0312 mg/L to 1024 mg/L. The effect of DMSO at the different concentrations was evaluated in an independent study, ruling out any possible toxicity for antibacterial assays.

### 4.2. Zeta Potential

Zeta potential was measured using a Zetasizer Nano ZS instrument (Malvern Instruments, Malvern, UK)Five measurements in automatic mode with a range between 10 and 100 cycles of each sample were made. Compounds were measured in distilled water at a concentration of 30 µM. The data were analysed using Malvern software.

### 4.3. Conductivity

Conductivity was measured using an Orion 3 Star instrument (Thermo Scientific, Waltham, MA, United States) and a conductivity cell ref. 013005MD. Compounds were measured in Milli-Q water at a concentration of 850 mg/L. As control samples, Milli-Q water (<1 µS/cm) and KCl 0.001 M (Scharlau, 147 µS/cm) were employed.

### 4.4. Bacterial Strains

For this work, a strain of the Gram-positive *Staphylococcus aureus* (CECT 240) was employed, which was provided by the Spanish Type Culture Collection (CECT) in lyophilised form.

### 4.5. In Vitro Antibacterial Activity Tests Against Planktonic Cells

The assay was based on the ISO 20776-1:2006 protocol. First, bacteria were grown in a Petri dish with PCA culture medium for 24 h at 37 °C. After inoculation, bacteria were incubated with biocides at each of the sixteen concentrations (0.0312 to 1024 mg/L), as well as with controls in sterile 96-well plates. The different concentrations of biocide were evaluated, as well as different controls to rule out any contamination or additional effects that could affect the correct reading of the plate (inoculum—sample without biocide; biocide—sample without inoculum; and culture medium—sample without inoculum and biocide) by triplicate. The plates were incubated for 24 h at 37 °C. The plates were analysed using an Ultra Microplate reader (BIO-TEK Instruments, model ELx808, Winooski, Vermont, United States), using a wavelength of 630 nm, at t = 0 h and t = 24 h. The results were collected to obtain the minimum inhibitory concentration (MIC) of the biocide. Subsequently, 5 µL of one of the repetitions of each biocide concentration and of the controls were deposited on a petri dish containing solid PCA medium. This test was performed in duplicate and incubated for 24 h to obtain the minimum bactericidal concentration (MBC) values. For the tests of antibacterial activity using 96-well plates, TSB (BD, ref. 211825) was used as culture medium. For the growth of bacteria in petri dish, Plate Account Agar (PCA) (Scharlau, ref. 01-161) was used as the culture medium.

### 4.6. In Vitro Antibacterial Activity Tests to Prevent S. aureus Biofilm Formation

The assay was based on the ISO 20776-1:2006 protocol. Bacteria were cultured by the streaking method in a PCA petri dish at 37 °C for 24 h and then some colonies were taken and added to a tube containing Bacto Tryptic Soy Broth (BD, ref. 211825) until 0.5 units of McFarland scale were obtained. The tube was incubated at 37 °C for 20 h to obtain the pre-inoculum. Afterwards, a dilution of 1:100 was made with the same medium (inoculum solution). An aliquot of 200 µL of inoculum solution was mixed with 50 µL of each of the 16 concentrations of the biocides and the controls in sterile 96-well plates and incubated at 28 °C for 10 h. The different concentrations of biocide were evaluated by triplicate and controls of inoculum, biocide and culture medium, were tested as well. Biofilm formation was measured as follows: first, the total absorbance of each well was measured using an Ultra Microplate reader (BIO-TEK Instruments, model ELx808, Winooski, Vermont, United States) at 630 nm. After that, the supernatant (planktonic cells) was transferred to new 96-well plates and the absorbance was measured again, to determine the minimum inhibitory concentration (MIC). In the first 96-well plate, the remaining biofilms were stained with 1% violet crystal in water for 15 min. After removing the excess dye with PBS (phosphate buffered saline, 10 mM, three gentle washing cycles), the plate was dried and 200 µL of acetic acid (33% water solution) was added to remove the dye inside the cells. The acetic acid solution was extracted from the well and deposited on another new 96-well plate in order to measure the absorbance of each well and determine the minimum biofilm formation inhibitory concentration (MBIC). In all cases, a 630 nm wavelength was used. The minimum bactericidal concentration for biofilms formation (MBC-B) was obtained using 5 µL of one of the replicates of each biocide concentration and controls for inoculating a petri dish with PCA medium. The plate was incubated for 24 h at 37 °C, and the assay was performed in duplicate.

## 5. Conclusions

Nanotechnology can be key for the prevention and treatment of bacteria biofilms. Carbosilane metallodendrimers merge the structural perfection and multivalency of the dendritic scaffold with exclusive biocide mechanisms generated by the metal complexes. A thorough structural design of these metallodendrimers can generate promising alternatives to traditional antibiotics. The dendrimer generation, the nature of the metal ion and its ligands, as well as the substituents on the chelating moieties are critical parameters to fine-tune the antibacterial activity not only against planktonic cells, but also against the resistant biofilms. We herein demonstrated (1) the need for a simple synthetic process, to reach a competitive antimicrobial candidate; and (2) the importance of adequately balancing the charge and the lipophilicity of the metallodrug to efficiently prevent biofilm formation. Carbosilane metallodendrimers present a brilliant future ahead with many possibilities to further improve and modulate their antimicrobial activity through structural design.

## Figures and Tables

**Table 1:** ^1^H-NMR shifts for selected signals in the new Ru(II) metallodendrimers and increase compared to the precursor dendrimers **I** and **II** [16].

Dendritic Fragment		δ (ppm)				
	Signal	1-Me	Δ	Signal	1-OMe	Δ
-Si(CH_2_)_3_N=CH-	-SiC*H_2_*	0.64	0.12	-SiC*H_2_*	0.62	0.10
	-C*H_2_*-	1.94/2.04	0.26/0.36	-C*H_2_*-	1.93/2.04	0.26/0.37
	-C*H_2_*N	4.24/4.68	0.62/1.06	-C*H_2_*N	4.24/4.67	0.63/1.06
	N=C*H*	8.62	0.30	N=C*H*	8.59	0.30
R-Py	*o-*	8.00	0.21	*o-*	7.75	0.28
	*m-*	9.30	0.82	*m-*	9.22	0.83
	*p-*	7.64	0.54	*p-*	7.34	0.54
	Me	2.61	0.25	OMe	4.05	0.20

**Table 2:** Bacteriostatic and bactericide effect of first-generation carbosilane Ru(II) metallodendrimers in *S. aureus* planktonic cells (MIC, MBC) and biofilm-forming cells (MBIC, MIC, MBC-B).

Metallodendrimer	Ligand/Counterion	Conductivity [µS/cm]	Zeta Potential [mV]	Planktonic		Biofilm		
				MIC [mg/L]	MBC [mg/L]	MBIC [mg/L]	MIC [mg/L]	MBC-B [mg/L]
**1-H**	Cym/Cl	160.2	61.2 ± 0.7	16	32	32	32	64
**1-Me**	Cym/Cl	137.5	59.5 ± 0.9	64	128	16	64	64
**1-OMe**	Cym/Cl	140.1	50.2 ± 0.7	32	128	32	64	128
**4-H** ^a^	Cp-PTA/Cl	^b^	25.6 ± 4.7	4	4	32	32	128

^a^ Previously published results [14]. ^b^ Not reported. Abbreviations: Cym, cymene; Cp, cyclopentadienyl; PTA, 1,3,5-Triaza-7-phosphaadamantane.

**Table 3:** Bacteriostatic and bactericide effect of first-generation carbosilane Cu(II) metallodendrimers in *S. aureus* planktonic cells (MIC, MBC) and biofilm-forming cells (MBIC, MIC, MBC-B).

Metallodendrimer	Counterion	Planktonic		Biofilm		
		MIC [mg/L]	MBC [mg/L]	MBIC [mg/L]	MIC [mg/L]	MBC-B [mg/L]
**2-H** ^a^	ONO_2_	4	8	4	8	8
**2-Me**	ONO_2_	64	128	32	64	128
**2-OMe**	ONO_2_	16	64	32	64	64
**3-H** ^a^	Cl	2	4	8	8	16
**3-Me**	Cl	16	64	32	64	128
**3-OMe**	Cl	8	16	8	16	32

^a^ Previously published results [14].

## Supplementary Materials

The following are available online at https://www.mdpi.com/article/10.3390/antibiotics10050589/s1, Figure S1: ^1^H-NMR and ^13^C-NMR of metallodendrimer **1-Me**; Figure S2: ^1^H-NMR and ^13^C-NMR of metallodendrimer **1-OMe**.
