# Supplementary material for: Insight on the Structure-to-Activity of Carbosilane Metallodendrimers in the Fight against Staphylococcus aureus Biofilms
Source: Antibiotics (Basel). 2021 May 17;10(5):589. doi: 10.3390/antibiotics10050589 (PMC8156651; doi:10.3390/antibiotics10050589)
Supplement: Supplementary file 1 [file antibiotics-10-00589-s001.zip › antibiotics-1211812-supplementary.pdf]

Submitted to Pharmaceutics

Article type: regular article

SUPPLEMENTARY INFORMATION

Structural design of carbosilane metallodendrimers to modulate the antibacterial effect against *Staphylococcus aureus* biofilms

Celia Llamazares[1], Natalia Sanz del Olmo[2], Juan Soliveri[1], F. Javier de la Mata[2,3,4], José Luis Copa-Patiño[1], and Sandra García-Gallego[2,3,4]*

[1] University of Alcalá, Department of Biomedicine and Biotechnology, Madrid, Spain; celia.llamazares@hotmail.es (C.L.); juan.soliveri@uah.es (J.S.); josel.copa@uah.es (J.L.C.-P.)

[2] University of Alcalá, Research Institute in Chemistry "Andrés M. del Río" (IQAR) and Faculty of Science, Department of Organic and Inorganic Chemistry, Madrid, Spain; n.sanzdelolmo@gmail.com (N.S.O.); javier.delamata@uah.es (J.d.M.); sandra.garciagallego@uah.es (S.G.G.)

[3] Networking Research Center on Bioengineering, Biomaterials and Nanomedicine (CIBER-BBN), Spain.

[4] Institute Ramón y Cajal for Health Research (IRYCIS), Spain.

* Correspondence: sandra.garciagallego@uah.es (S.G.G.).

Table of Contents

Figure S1. ^{1}H-NMR and ^{13}C-NMR of metallodendrimer **1-Me**……………………………………………………….2

Figure S2. ^{1}H-NMR and ^{13}C-NMR of metallodendrimer **1-OMe**………………………………….……………….3

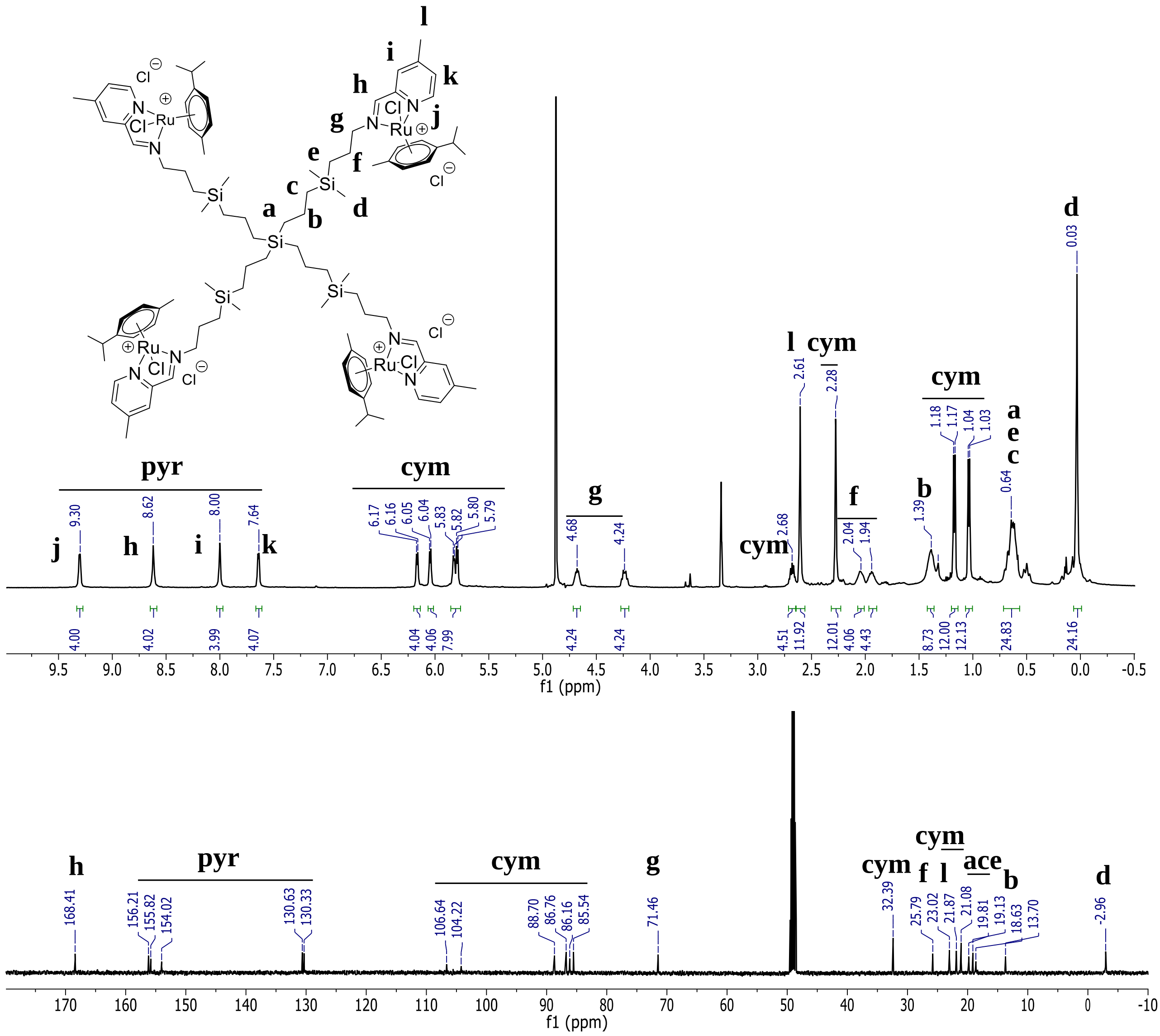

Figure S1. ^{1}H-NMR and ^{13}C-NMR (500MHz, CD_3OD) of metallodendrimer **1-Me**.

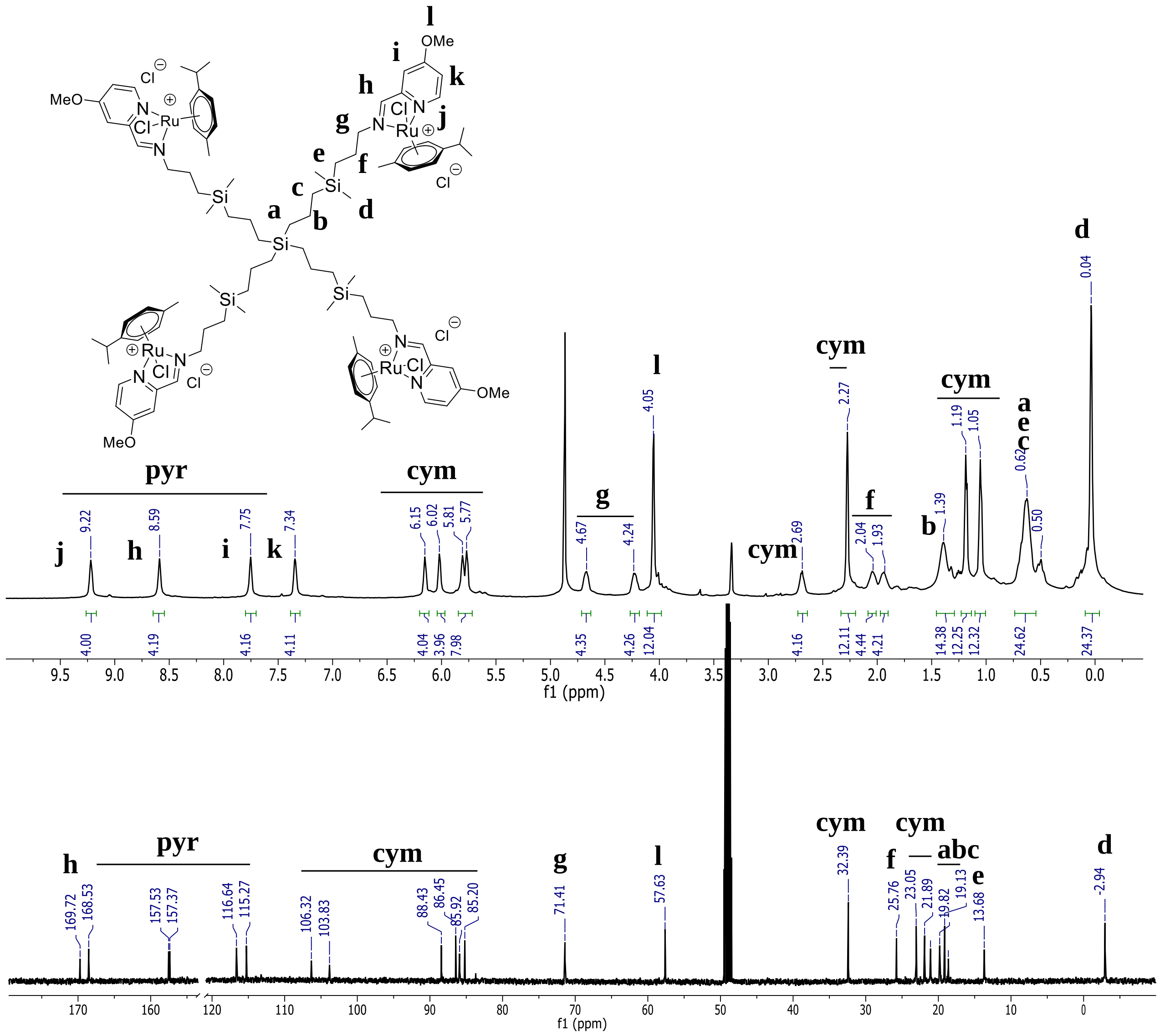

Figure S2. ^{1}H-NMR and ^{13}C-NMR (500MHz, CD_3OD) of metallodendrimer **1-OMe**.
